# Diseases of the Nervous System

**Published:** 1892-12

**Authors:** 


Diseases of the Nervous System. By Jerome K. Bauduy, M. D., LL. D., Professor of the Diseases of the Mind and Nervous System, and of Medical Jurisprudence, Missouri Medical College, St. Louis; late Physician-in-Chief to St. Vincent's Institution for the Insane; Corresponding Member of the New York Society of Neurology and Electrology; formerly Consulting Physician of the St. Louis County Lunatic Asylum; Member of the New York Medico-Legal Society, etc. Second edition. Philadelphia: J. B. Lippincott Co. 1892.

On reading the title page of this book, one would naturally expect to find a treatise on diseases of the peripheral nerves, spinal cord, and brain, but such is not the case. The author, in a series of lectures, discusses in a pleasing, painstaking way some pathological conditions of the meninges, and more thoroughly, perhaps, the various forms of insanity. In justice to the author, however, he promises in the preface to add another volume, in which will be discussed the diseases of the brain, cord, and nerves. It would have been well to have designated this as Volume I., either on the back or on the title page, so as to avoid misunderstanding.

The subject matter is presented in excellent form, and the references to American and foreign authors shows the author to have been a close student of neurology. In speaking of the abbreviated terms "dura" and "pia," the writer remarks, in a foot-note, that at a *recent* congress of European neurologists it was decided to drop the word mater, using only the words dura and pia. Perhaps the writer is not aware of Professor Wilder's efforts in behalf of a revised terminology; but the reviewer has listened many times to Professor Wilder's exhortation for shorter and more appropriate scientific terms. The author's views on insanity are extremely well described, particularly the chapters on Epileptic Insanity, which treat of the subject in a careful and forcible manner.

The author pays considerable attention to treatment, and herein he differs from most writers on neurology. Neurologists, as a rule, pay little attention to therapeutics, but limit themselves to the pathology and symptomatology of the disease in question.

On the whole, this book is worthy of careful reading by the specialist and general practitioner, but hardly can be recommended to students. The press-work is excellently done. W. C. K.

